# Role of bone scan in diagnosis of calciphylaxis: A review

**DOI:** 10.1016/j.jdin.2023.09.010

**Published:** 2023-10-23

**Authors:** Morgan Groover, Fnu Nutan

**Affiliations:** Department of Dermatology, University of Massachusetts Chan Medical School, Worcester, Massachusetts

**Keywords:** bone scan, bone scintigraphy, calciphylaxis, consultative dermatology, hospital medicine, inpatient

**Figure:**
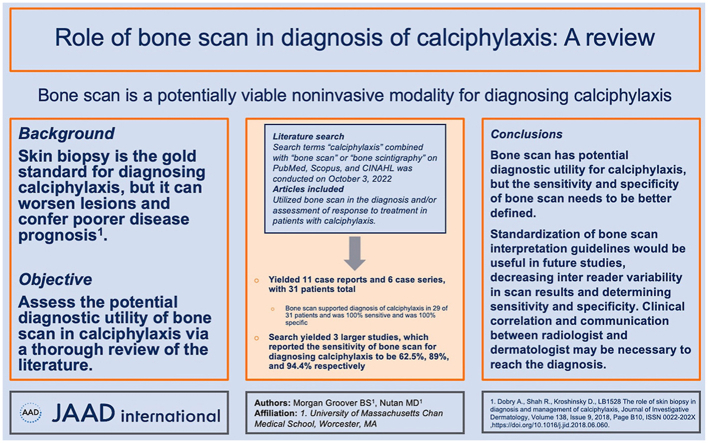

*To the Editor:* Skin biopsy is the gold standard for diagnosing calciphylaxis, but it can worsen lesions and confer poorer disease prognosis.^1^ Several reports have considered technetium-99m methyl diphosphonate bone scan as a noninvasive diagnostic alternative to skin biopsy in calciphylaxis, but data regarding its sensitivity and specificity are limited. Our goal was to assess the potential diagnostic utility of bone scan in calciphylaxis via a thorough review of the literature.

A comprehensive search of PubMed, Scopus, and Cumulative Index to Nursing and Allied Health Literature was conducted on October 3, 2022. Search terms included “calciphylaxis” combined with “bone scan” or “bone scintigraphy.” No time constraints were placed on this search. Articles were included if they used bone scan in the diagnosis and/or assessment of response to treatment in patients with calciphylaxis.

The search yielded 11 case reports and 6 case series, with 31 patients in total. Patient characteristics and details of the diagnostic modalities used in calciphylaxis diagnosis are given in Table I^2^, ^3^, ^4^, ^5^ (additional references are present in the Supplementary Material, available via Mendeley at https://data.mendeley.com/datasets/88fkkbwvkt/1). All but 1 patients had renal disease, and most patients had several other comorbidities. Bone scan supported the diagnosis of calciphylaxis in 29 of 31 patients, with 100% sensitivity and specificity, respectively. Among the 15 cases in which both bone scan and biopsy were performed, 3 patients had an equivocal biopsy with positive bone scan, and there were no cases in which biopsy and bone scan results were contradictory. Additionally, the search yielded 3 larger studies from China, United States, and Canada, which reported the sensitivity of bone scan for diagnosing calciphylaxis as 62.5%, 89%, and 94.4%, respectively (Table II and Supplementary Material). One of the studies had a 97% specificity.

**Table I:** Characteristics of patients with calciphylaxis and bone scan

Patient No.	Patient age (y)	Patient sex	Bone scan results	Biopsy supports the diagnosis of calciphylaxis	Other imaging modality	Source
1	65	Male	Positive	Yes	CT	Itani et al, 2016
2	66	Female	Positive (metastatic pulmonary calciphylaxis)	Equivocal	Magnetic resonance imaging, CT	Santos et al, 2021
3	40	Female	Positive	Yes		Raduka et al, 2018
4	32	Male	Positive	Yes		Di et al, 2020
5	26	Female	Positive (metastatic pulmonary calciphylaxis)	NA	SPECT-CT, low-dose CT	Martineau et al, 2017
6	75	Female	Positive	NA	SPECT-CT	Norris et al, 2005
7	52	Female	Positive	Yes	X-ray	Han et al, 2007
8	19	Female	Positive	Yes (lung and breast biopsy)	CT	Shen et al, 2017
9	59	Female	Positive	NA	X-ray	Wheeler et al, 2015
10	51	Female	Positive	NA		Soni et al, 2008
11	60	Female	Positive	NA		Cosmin et al^5^, 2005
12	73	Female	Positive	Yes	X-ray	Fine et al, 1995
13	59	Female	Positive	NA		Fine et al, 1995
14	46	Female	Positive	NA		Fine et al, 1995
15	56	Female	Positive	NA	X-ray	Fine et al, 1995
16	41	Female	Positive	NA		Fine et al, 1995
17	NA	NA	Positive	Equivocal		Alniemi et al^3^, 2020
18	NA	NA	Positive	Equivocal		Alniemi et al^3^, 2020
19	NA	NA	Negative	No		Alniemi et al^3^, 2020
20	NA	NA	Negative	No		Alniemi et al^3^, 2020
21	21	Male	Positive	No	X-ray	Araya et al^4^, 2006
22	12	Male	Positive	Yes		Araya et al^4^, 2006
23	21	Female	Positive	NA		Araya et al^4^, 2006
24	64	Female	Positive	Yes		Maeda et al, 2007
25	21	Female	Positive	Yes		Maeda et al, 2007
26	45	Female	Positive	NA		AlBugami et al^2^, 2013
27	65	Male	Positive	NA		AlBugami et al^2^, 2013
28	48	Female	Positive	NA		AlBugami et al^2^, 2013
29	61	Male	Positive	NA		AlBugami et al^2^, 2013
30	44	Female	Positive	NA		Gripp et al, 2015
31	54	Female	Positive	NA		Gripp et al, 2015

*CT*, Computed tomography; *NA*, not applicable; *SPECT*, single-photon emission computed tomography.

**Table II:** Sensitivity results from larger studies

Study type	Epidemiological	Case control	Case control
Study location	China	USA	Canada
Total patients diagnosed with calciphylaxis	48	18	36
Bone scan sensitivity	62.50% (30/48)	89% (16/18)	94.40% (34/36)
Bone scan specificity	NA	97% (negative in 30/31 controls)	NA
Biopsy sensitivity	64.71% (performed in 34/48 cases)	NA	100% (performed in 4/36 cases)
Source	Liu et al, 2022	Paul et al, 2017	Fine et al, 2002

*NA*, Not applicable.

The individual cases and larger studies presented herein demonstrate that bone scan is a potentially viable noninvasive modality for diagnosing calciphylaxis. The 100% sensitivity of bone scan demonstrated by the individual cases is promising, but there is a limitation that published cases were likely biased toward patients in whom bone scan detected disease. The larger studies reported broader ranges of bone scan sensitivity for calciphylaxis, likely reflecting variability among readers. This is unsurprising considering that there is no grading system for radiologist interpretation of bone scan in calciphylaxis. Standardization of bone scan interpretation guidelines would be useful in future studies, decreasing interreader variability in scan results and determining sensitivity and specificity. Moreover, clinical correlation and collaboration between the dermatologist and the nuclear medicine physician may still be necessary to reach a diagnosis considering the differential diagnosis of dermatologic conditions associated with delayed radiotracer clearance from soft tissues detected on bone scan.

Additionally, bone scan has the potential to detect systemic calciphylaxis in cases in which it may not be clinically evident, to monitor response to treatment, and to potentially screen for calciphylaxis in high-risk groups. Given the morbidity and increasing incidence of calciphylaxis and the need for a diagnostic algorithm, the role of bone scan in disease management needs to be defined better.

## Conflicts of interest

None disclosed.
